# Significance of *Shewanella* Species for the Phytoavailability and Toxicity of Arsenic—A Review

**DOI:** 10.3390/biology11030472

**Published:** 2022-03-18

**Authors:** Aminu Darma, Jianjun Yang, Peiman Zandi, Jin Liu, Katarzyna Możdżeń, Xing Xia, Ali Sani, Yihao Wang, Ewald Schnug

**Affiliations:** 1 Institute of Environment and Sustainable Development in Agriculture, Chinese Academy of Agricultural Sciences, Beijing 100081, China; aidarma.bio@buk.edu.ng (A.D.); xiaxing@caas.cn (X.X.); wangyihao1996116@126.com (Y.W.); 2 Department of Biological Sciences, Faculty of Life Science, Bayero University, Kano 700006, Nigeria; asani.bio@buk.edu.ng; 3 International Faculty of Applied Technology, Yibin University, Yibin 644600, China; peiman.zandi@yibinu.edu.cn; 4 College of Agronomy and Biotechnology, China Agricultural University, Beijing 100094, China; jliu207@cau.edu.cn; 5 Institute of Biology, Pedagogical University of Krakow, Podchorążych 2 St., 30-084 Krakow, Poland; kasiamozdzen@interia.pl; 6 Department of Life Sciences, Institute for Plant Biology, Technical University of Braunschweig, 38106 Braunschweig, Germany

**Keywords:** arsenic, detoxification, dissimilatory arsenic-reducing bacteria (DARB), *Shewanella* species, transformation, remediation

## Abstract

**Simple Summary:**

The availability of some toxic heavy metals, such as arsenic (As), is related to increased human and natural activities. This type of metal availability in the environment is associated with various health and environmental issues. Such problems may arise due to direct contact with or consumption of plant products containing this metal in some of their parts. A microbial approach that employs a group of bacteria (*Shewanella* species) is proposed to reduce the negative consequences of the availability of this metal (As) in the environment. This innovative strategy can reduce As mobility, its spread, and uptake by plants in the environment. The benefits of this approach include its low cost and the possibility of not exposing other components of the environment to unfavourable consequences.

**Abstract:**

The distribution of arsenic continues due to natural and anthropogenic activities, with varying degrees of impact on plants, animals, and the entire ecosystem. Interactions between iron (Fe) oxides, bacteria, and arsenic are significantly linked to changes in the mobility, toxicity, and availability of arsenic species in aquatic and terrestrial habitats. As a result of these changes, toxic As species become available, posing a range of threats to the entire ecosystem. This review elaborates on arsenic toxicity, the mechanisms of its bioavailability, and selected remediation strategies. The article further describes how the detoxification and methylation mechanisms used by *Shewanella* species could serve as a potential tool for decreasing phytoavailable As and lessening its contamination in the environment. If taken into account, this approach will provide a globally sustainable and cost-effective strategy for As remediation and more information to the literature on the unique role of this bacterial species in As remediation as opposed to conventional perception of its role as a mobiliser of As.

## Highlights

- Dissolution of As-bearing minerals by dissimilatory arsenic-reducing bacteria (DARB) releases As into the environment.
- Arsenic toxicity has a wide range of effects on plants and other environmental components.
- *Shewanella spec.*-mediated As detoxification could be a valuable tool for As remediation.
- *Shewanella spec*.-mediated methylation could limit the availability of toxic arsenicals in the environment.
- Flavins secreted by *Shewanella oneidensis MR-1* facilitate As sequestration and detoxification.

## 1. Introduction

The wide distribution and occurrence of arsenic (As) throughout the earth’s crust arise due to its vast natural and anthropogenic sources. It is mainly found in trace amounts with an average crustal concentration of 5.5 mg/kg [1]. Rock phosphates have As concentrations in the same range, with averages of 3.33 mg/kg for igneous and 5.59 mg/kg for sedimentary phosphates [2]. Thus, fertilising with mineral phosphate fertilisers in plant production contributes significantly to the As load in agricultural soils, which is estimated for Germany alone to be 40–73 t/yr [3]. After 70 years of intensive agriculture, fertiliser-derived As accounts for up to 1.4% of average background concentrations of As in soils [3].

Being among the most toxic heavy metals [4,5,6], its related pollution has become a global environmental concern for groundwater and agricultural soils, causing detrimental effects to plants and other living organisms [7,8]. Natural and anthropogenic processes are the leading causes of As mobilisation and release into the environment [9]. The natural rise and release of As in groundwater and sediment are always associated with the bio-reduction of As-bearing minerals [10,11,12]. In a natural setting such as underground water, sediments, and soil, As can be found in association with oxides of iron (Fe) and microbial consortia, which could influence its speciation, mobility, toxicity, and availability.

Fe is the fourth most ubiquitous element in the Earth’s crust and can be found in almost any sediment, soil, surface, or subsurface water [13,14,15]. Although it has a variable range of oxidation states (−2 to +6) [16], in natural settings, it exists as either ferrous iron (Fe(II)) or ferric iron (Fe(III)). Because Fe(II) is notably more soluble than Fe(III), it is comparatively more abundant in bioavailable forms [13,17]. The weathering of rocks [18], reduction of numerous Fe(III) ligand complexes [19], and reduction of Fe(III)-bearing minerals such as ferrihydrite, goethite, hematite, magnetite, and lepidocrocite by dissimilatory iron(III)-reducing bacteria (DIRB) [20,21,22,23,24,25,26,27,28] are some of the processes that could result in the formation of Fe(II) in the environment. Interestingly, the geochemical cycling of Fe and Fe-bearing mineral dissolution affects the availability, mobility, sequestration, decomposition, and remediation of the associated metals such as cadmium (Cd), chromium (Cr), and As [21,29,30,31,32,33,34,35,36,37,38].

Interactions between some microorganisms and Fe-bearing minerals in the environment result in the change of speciation and toxicity of some heavy metals. For instance, dissimilatory reductive dissolution of Fe and As result in a significant increase in the availability of Fe(II) and As(V) in the solid and aqueous phases [39,40]. This process could lead to the interconversion and mobilisation of inorganic arsenic, i.e., As(III) and As(V), and methylated arsenicals such as monomethylated arsenic (MMA), dimethylated arsenic (DMA), and trimethylated arsenic (TMA) [41,42]. Similarly, reductive dissolution of accessible As(V) by dissimilatory arsenic-reducing bacteria (DARB) facilitates the mobilisation of toxic As(III) [9,43]. Notably, reduction of As-containing Fe minerals does not always result in toxic As species mobilisation, but rather in the availability of As(V), which, despite being less toxic than As(III), is still toxic to some organisms [21,24]. However, depending on biological and chemical conditions, the stability of As(V) may be disrupted, making As(V) and As(III) interchangeable, thus, allowing As(III) to be freely available in the environment, resulting in As toxicity in and around the affected environment.

*Shewanella oneidensis* MR-1, a member of the *Shewanella* genus, is one of the most important bacterial strains that facilitates Fe(II) mobilisation by reductive dissolution of Fe(III) [25,30,44,45]. This bacterium is common in aquatic habitats and can be found in various ecological niches [46]. This ecological distribution has increased its ability to withstand As toxicity through diverse physiological mechanisms such as methylation [42,47] and detoxification [48,49]. Additionally, several of this bacterium’s cellular secretions, such as flavin, which primarily serves as electron shuttles for Fe(III) reduction to Fe(II) [50,51,52], have contributed to organo-arsenical detoxification [53] and production of iron plaques, which have a high affinity to arsenic and thus contribute to the immobilisation of the surrounding As [38]. Even though the primary function of MR-1 is to promote the reduction of Fe(III) to Fe(II), emerging research suggests that the bacterium may play a role in both the sequential mobilisation and detoxification of arsenic, indicating its importance in resolving issues related to As mobility and contamination. Recently, the catalytic methylation of As by some species of bacteria using methyltransferase (*arsM*) resulted in a decline of toxic arsenicals in the environment [54].

Presently, there is limited information on how the interplay between electron shuttles, flavin secretion, and the reduction of Fe in As-bearing minerals by the *Shewanella* genus, specifically MR-1, contributes to the design of in situ As remediation strategies.

This article explores arsenic toxicity and major remediation approaches with their drawbacks considering increases in information from the past decades. Given the role of Fe as a key component of the environment, as well as the numerous processes and mechanisms that drive its biogeochemical cycling, its impact, as well as the influence of MR-1, as a model dissimilatory Fe-reducing bacterium, which resulted in the availability and mobility of Fe and As in the environment, is discussed. Finally, the potential of MR-1 as a tool for As toxicity management rather than only Fe-mobilising bacteria is highlighted, together with specific gaps and future directions that will lead to achieving such a breakthrough.

## 2. Arsenic Species and Their Behaviour in the Environment

Arsenic is a member of group Va of the periodic table and is classified as a metalloid that readily reacts with numerous elements [24]. It exists in the environment in different oxidation states as Arsine (As(III^−^)), Arsenic (As(0)), Arsenate (As(V)), and Arsenite (As(III)) [24,55].

**Figure 1:**
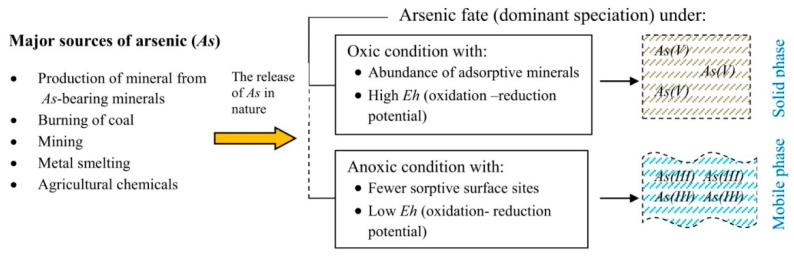
Behaviour of arsenic (As) from different sources under oxic and anoxic conditions. In oxic sub-surface systems with a high *Eh* (oxidation-reduction potential) and abundance of adsorptive minerals, As(V) tends to be immobilised in the solid phase. Under anoxic conditions (low *Eh*), As(III) slightly binds to fewer sorptive surface sites and therefore, distributes into the aqueous phase and is thus more mobile (and notably more toxic) than As(V). Courtesy of word diagram: Aminu Darma and Peiman Zandi.

The inorganic oxyanions of As(III) or As(V) are the most typical soluble arsenic species that exist mainly in natural water [56]. By contrast, dimethyl arsenic acid (DMAA) and monomethyl arsenic acid (MMAA) mainly comprise the organic arsenic species [42,55]. The species of As(III) include AsO_3_^3−^, HAsO_3_^2−^, H_2_AsO_3_^−^, and As(OH)_3_, while H_3_AsO_4_, H_2_AsO_4_^−^, HAsO_4_^2−^, and AsO_4_^3−^ constitute the As(V) species [57]. With respect to complex formation, As(V) can form complexes with sulfides and a ternary complex with polyvalent cations, whereas As(III) assists in complex formation with some metals and other natural organic materials in the environment [58,59]. Depending on the prevailing conditions, the availability of the inorganic As species differs (Figure 1). As (III) dominates under reducing anaerobic conditions while As(V) is dominant under oxygen-rich aerobic conditions and is often more associated with the solid phase [12,23,24]. However, environmental factors such as the redox potential (*Eh*), organic matter, and pH, among others, influence As speciation, mobility, and toxicity because studies have already shown variation in the behaviour of these inorganic As species in response to changes in some of these ecological variables [23,24,60,61,62].

### Influence of Environmental Factors on the Mobilisation of As

The mobility of As species and their overall availability is strongly linked to environmental factors. These factors, as described in several research studies include but are not limited to the redox potential (*Eh*), pH, microbes, and natural organic matter [63,64]. In most natural settings, As is associated with mineral compounds such as realgar (As_4_S_4_), arsenopyrite (FeAsS), and orpiment (As_2_S_3_) [65] and can be closely associated with metals such as Fe, Pb, Cd, and Ni, among others [64,66]. Additionally, some bacteria, particularly IRB, utilise the As transformation process for energy generation and or as a precursor of phosphorus (P) for the synthesis of adenosine deoxyribonucleic acid (DNA) triphosphate (ATP), ribonucleic acid (RNA), and phospholipids [64,67]. Therefore, variation or alteration of the environmental factors affects the speciation and mobility of As and often influences the activity of the associated microbes, which in turn affect the solubility, availability, and toxicity of As in the environment [63]. The detailed information on the influence of environmental variables on As mobilisation has been explained by [64].

## 3. Arsenic Toxicity and Its Consequences

Animals and plants are susceptible to As’s toxic and carcinogenic effects [24]. Generally, organic As species are less harmful to living organisms than inorganic As forms [25,68,69,70]. The risk assessments of this metalloid in food and drinking water were comprehensively reported and documented in an article by Rasheed et al. [71] and Hirano [42]. Equally interesting is that, according to historical evidence, As species toxicity persists because it can be methylated, and its oxidation state changes during metabolism [42].

The most important and well-known toxic environmental species are As(V) and As(III) [71,72,73]. These As species are recognised to cause skin disorders, lesions in peripheral blood, and cancers in animals, particularly humans [71]. Other categories of cancers associated with chronic exposure to these species through drinking contaminated water include urinary bladder, lung, skin (primarily squamous cells), and possibly liver, kidney, and prostate cancers [42,74].

From another perspective, As toxicity in plants causes cytotoxicity and the production of reactive oxygen species (ROS), which include the hydroxyl radical, superoxide anion, and hydrogen peroxide, which cause genomic instability and inhibit the activities of some cellular enzymes [75]. Additionally, an increase in As contents in plants is associated with decreased plant growth and yield [34,76]. Moreover, other physiological and several morphological changes such as a decrease in the germination rate, reduction in the number of leaves, plasmolysis of the root cells, necrosis of leaf tips, leaf wilting, disruption of the cellular membrane structure, inhibition of photosynthesis, and disruption of growth patterns are commonly associated with As toxicities in plants [76,77]. Furthermore, when absorbed by plants, As may become a part of the food chain, posing a threat to food safety [78,79].

## 4. Mitigation and Remediation Strategies for Arsenic

Arsenic is mainly released into the environment due to volcanic and industrial activity. Mining, non-ferrous metal smelting, and fossil fuel combustion are major anthropogenic activities that result in As contamination of water, air, and soil [9,80,81,82]. Several reports have also proved that using As to preserve wood has resulted in As-related environmental contamination [83]. As previously stated, As contamination causes numerous health and environmental problems. Thus, it is crucial to understand and devise remediation methods to immobilise and/or reduce its availability in the environment. Several approaches were developed and evaluated for the remediation of As contamination in the environment. These strategies are grouped as physical, chemical or biological methods [55]. Furthermore, new and emerging treatments technologies have been thoroughly assessed and are now being evaluated. Some of these strategies were further studied in recent studies [84,85]. Primarily, the broad and basic strategy in controlling As contamination is the transformation of toxic As to the less harmful As species. Further, depending on the geochemistry of the contamination, the physical and chemical conversion in this approach involves the use of strong oxidants, such as hydrogen peroxide, potassium permanganate, or ozone as a strategy [9]. In this conventional approach, coagulation, filtration, inverse osmosis, and ion exchange are extensively involved, which are expensive, labour intensive, and are capable of further instigating undesirable consequences such as environmental pollution and so on due to the chemicals involved [86,87,88]. For this and other reasons, biological methods focused on plant- and microbial-based approaches were given more attention because they are safer, better, and economically viable solutions for the environment.

Phytoremediation plays an essential role in environmental pollution mitigation and management. This is because plants are both inexpensive and environmentally friendly for heavy metal decontamination of soil [34,88,89,90]. The plants usually involved in this process are called hyperaccumulators, as they can absorb and accumulate the metals in their upper parts [90,91]. For example, *Epilobium fragilis* was reported to accumulate As from contaminated soil [92]. Also, in a study using native plants, Yildirim and Sasmaz [90] observed that *Anchusa arvensis*, *Phlomis* sp., *Glaucium flavum*, and *Verbascum thapsus* demonstrated the capacity of cleaning and remediating soil affected by As pollution. Recently, Patel et al. [77] provided an account and list of various tolerant plants and potential contributors for As phytoremediation. Despite the widespread use of several plants in the control and management of As toxicity, the method is time-consuming and requires more effort in screening suitable prospects, as not all plants can accumulate heavy metals [93,94]. Furthermore, wider consideration is essential regarding the ion uptake mechanism of the prospective plants relative to their morphological, physiological, anatomical, and genetic traits [90,95]. Therefore, alternative strategies mediated by microbes (fungi and bacteria) serve as an additional helpful approach to mitigate and control heavy metal pollution in soil and water [21,96,97].

Heavy metal remediation by microbes, especially bacteria, provides some cushion effects from some of the challenges of other remediation approaches. This is because bacterial remediation is relatively inexpensive, effective, and eco-friendly [98]. DIRB are among the many bacteria used in this strategy because of their unique ability to use a wide range of electron acceptors in their respiration process [99,100]. This form of remediation is typically employed *ex-situ* (in the areas where contamination occurs) or in situ, for example, in storage tanks [100]. Because of differences in the oxidation state, solubility, and availability of each heavy metal and their varying behaviours in relation to prevailing environmental factors, bacterial heavy metal remediation is not uniform across all heavy metals [99,100].

For example, some *Shewanella* species have demonstrated the capacity to lessen the mobility of uranium (U) in groundwater as a result of the transformation of U(VI) to insoluble U(IV), leading to a decrease in the spread of U contamination from groundwater [100]. Bencheikh-Latmani et al. [101] reported how the cleanup effort for Cr has been greatly assisted by some *Shewanella* species through the formation of solid oxides during the reduction of soluble Cr(VI) to Cr(III). Conversely, methylmercury, a mobile bioaccumulative environmental toxin, has been reported to be formed as a result of *S. oneidensis* MR-1 reducing ionic mercury [Hg(II)] to elemental mercury [Hg(0)] [99,102]. Furthermore, catalytic reduction of As(V) to a mobile and more toxic As(III) facilitated by some *Shewanella species* commonly results in the mobilisation of As species that are toxic in the environment [64,103].

Having described the key role of *Shewanella species* in the facilitation of direct or indirect mobilisation of environmental As species, its potential remediation role could be best perceived from its contribution to the detoxification of some As species and/or sequestration of the mobilised As through flavin-mediated iron plaque formation [104,105,106]. These various processes are explained in detail in the upcoming sections of this article.

## 5. Iron and Its Impact on the Fate of Arsenic

Iron (Fe) is the fourth most available element on earth [107] and the lithosphere’s second most abundant transition metal element [108]. Its oxides comprise Fe and oxygen, including ferrihydrite, lepidocrocite, goethite, and magnetite [33,109,110,111]. These oxides are abundantly found in rocks, soil, sediments, water, and various minerals formed under different conditions [112]. Fe primarily exists in two redox states in aquatic settings, i.e., Fe(III) and Fe(II). At neutral pH and in the absence of complexing ligands, Fe(III) exists in the Fe(III) (hydr)oxide solid-state [37,113]. However, in a situation where a terminal electron acceptor and molecular oxygen (O_2_) are lacking, Fe(III) (hydr)oxides contribute to microbial anaerobic respiration where they serve as terminal electron acceptors [113,114].

Generally, under oxic conditions, toxic metals such as lead (Pb), Cd, Cr, and As are integrated and incorporated into amorphous or crystalline Fe(III) mineral phases [29,115,116,117,118]. Secondary Fe(III)- and Fe(II)-bearing minerals such as hematite and magnetite are closely associated with these metals [116,118,119]. Many studies have long established that, among these toxic heavy metals, As is adsorbed rather strongly to Fe-oxides [96,120,121], making it the primary reservoir and transporter of As in soil and aquifers [116,122].

**Figure 2:**
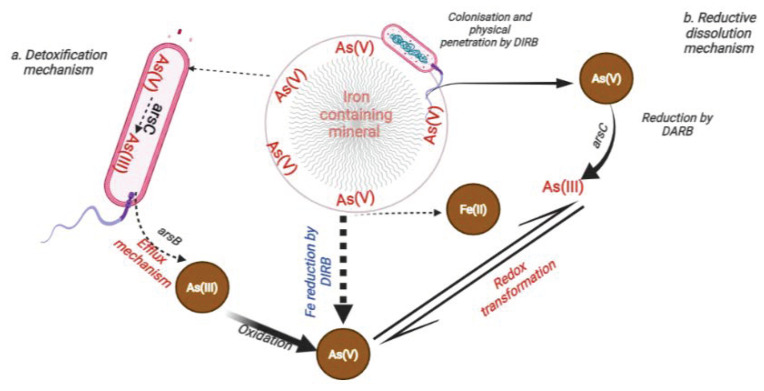
Conceptual model explaining (*a*) Detoxification pathway by which bacteria protect their cells from the toxic effect of arsenic metal from the environment by blocking the membrane channels through which toxic substances enter the cell. The bacteria transform As(V) to As(III) in the cell before its active exclusion from the cell via a membrane pump, which is controlled by the *arsB* gene (*b*) Reductive dissolution involves colonisation and physical penetration of primary minerals by As/Fe-reducing bacteria, resulting in the release of As(V) and Fe(II). Further action of dissimilatory arsenic-reducing bacteria through a cytoplasmic reductive gene (*arsC*) facilitates the reduction of As(V) to As(III). Oxidisation of released As(III) usually occurs in a different phase, leading to an increase in the abundance of As(V) in the surroundings. Courtesy of conceptual model: Aminu Darma.

It has been reported that the reductive dissolution of hydrous Fe-oxides and the subsequent release of related As can occasionally result in high As concentrations in subsurface waters [24,27]. Similarly, Fe(II) released from As-bearing minerals is an important catalyst linked with the mobilisation of As in the environment [123,124]. Despite the complexity of the process, many bacterial strains interact and dissolve the surface layers or structural regions of these minerals (Figure 2), resulting in Fe(II) generation [112,125,126]. This reduction of Fe(III) and subsequent Fe(II) release are strongly associated with As(V) dislodgement and its availability in the soil solution and the environment [13,118,123,127,128,129].

### Effect of Dissimilatory Iron-Reducing Bacteria’s Transformation of Iron on the Fate of Arsenic Soils

In general, dissimilatory metal-reducing bacteria (DMRB) oxidise organic matter, including organic contaminants and hydrogen gas (H_2_) in anoxic environments, and then transmit the released electrons to solid-phase minerals containing oxidised metal ions, such as manganese (Mn(IV)) and ferric iron (Fe(III)) for anaerobic respiration [38]. These microorganisms are commonly involved in the degradation/corrosion of organic matter [130] and metals [131,132], e.g., Pb, Cd, and As, in surface and sub-surface environments such as sediments of rivers, soils, lakes, and oceans. Additionally, the bacteria involved have the potential to dramatically alter the geochemistry of the surrounding Fe mineral-containing As in groundwater and sediments, resulting in As contamination of drinking water sources, diseases, poisoning, and human health disruption [39,133,134]. Numerous bacterial strains were identified to be involved in the reduction and transformation of Fe, particularly in the As(V)-bearing Fe(III) mineral assemblage [104,135,136]. These bacteria include dissimilatory iron-reducing bacteria (DIRB) and dissimilatory arsenic-reducing bacteria (DARB)*,* comprising *Shewanella oneidensis* MR-1 and *Geobacter metallireducens* GS-15 and *Geobacter* sp. OR-1 & *Bacillus* sp. M17-15, respectively.

Based on published evidence, *Geobacter metallireducens* GS-15 and *Shewanella oneidensis* MR-1 are among the early DIRB strains that promote Fe(III) reduction [64,107]. The role of *Shewanella oneidensis* MR-1 in this phenomenon was discovered when it was isolated from the silt of a freshwater lake; since then, its activity has been linked to intracellular oxidation of organic materials to extracellular Fe(III)(hydr)oxide reduction [137,138]. Similarly, numerous researchers have reported the influence of this strain on the geochemical cycling of organic matter and other metals, including Fe, As, etc. [137,139]. From the moment of such discovery to date, evidence from literature has consistently established the role of *Shewanella oneidensis* MR-1 as a typical DIRB that promotes the redox transformation of Fe(III)-oxides either directly or indirectly [49,123,140,141,142,143]. Therefore, it is assumed that the fate and biogeochemical transformations of Fe and associated metals, e.g., As, are strongly linked [118,144]. Nevertheless, in most situations, the transformation of Fe(III) to Fe(II) by DIRB only serves as a source of As(V) in the environment but does not guarantee As(V) solubilisation or subsequent As(III) mobilisation. This peculiarity was confirmed by Cummings et al. [145] in an effort to evaluate the role of *Shewanella alga* BrY in the reductive dissolution of scorodite. The study results revealed the presence of As(V), but As(III) was below the detection limit. Lopez-Adams et al. [124] recently reported the absence of As(III) in a supernatant during extracellular reduction of Fe(III)-(oxyhydr)oxide by *Shewanella* sp. cultures through nanoscale secondary ion mass spectrometry (NanoSIMS) imaging. These findings indicated that Fe(III) reductions by *Shewanella* sp. can cause As contamination but may not always result in As(III) mobilisation. Hence, the process can be considered an essential route for As(V) mobilisation into the environment.

At circumneutral pH and under oxic conditions, insoluble Fe(II) (hydr)oxides are produced from Fe(III) reduction [113]. An increase in the Fe(II) concentration in the environment is commonly interpreted as an indicator of reductive mineral dissolution by DIRB. Thus, in a complex environment where As is sorbed to Fe-bearing minerals, DIRB dissolution often promotes As mobilisation, resulting in As contamination of such an environment. Notably, readsorption of As often occurs because of the recrystallisation of more stable Fe(II)-oxides [113,127] or aggregation of Fe(II) to secondary minerals such as vivianite and magnetite, which subsequently result in arsenic re-sequestration from the environment [146,147,148].

## 6. The Impact of Bacteria on Arsenic Transformation and Cycling

Microorganisms have evolved diverse strategies to defend themselves against As through time. Fungi, for example, were known to protect themselves through a methylation process in which MMA or DMA was used. However, bacteria and the archaeal domain often produce volatile methylated arsines, facilitating the elimination of As in the environment [48,149]. In the process of tolerating and enduring metal toxicity, some bacteria precipitate, sequester, or even alter these metals’ oxidation state, and these processes often result in mobilisation or remediation of the metal in the environment [109]. Although As is generally toxic to life, many bacterial strains occasionally derive their energy for growth through As metabolisation [39,150], and this is achieved through the development of complex mechanisms of arsenate reduction, arsenite-oxidation, and/or As(V) resistance, a process which lessens the quantity of As entering the microbial cell [9,151,152]. Others can also use As compounds as donors or acceptors of electrons through As detoxification mechanisms [144,153]. Furthermore, metabolic and detoxification processes such as reduction, oxidation, methylation, and demethylation [154] cause the dissolution of As reservoirs, resulting in As mobilisation into the surrounding environment. Examples of such bacteria include the predominant DARB, heterotrophic arsenite-oxidising bacteria (HAO), chemoautotrophic arsenite-oxidising bacteria (CAO), and arsenate resistant bacteria [39,119,155].

### 6.1. Influence of DARB on the Transformation and Mobilisation of Arsenic in the Environment

Bacteria, especially DARB, interact with less toxic As(V), and by this process, the bacteria make As(III) available via two steps (Figure 2). Firstly, by reducing As(V) to As(III) using organic material as a prime source of energy for growth and secondly, through the detoxification mechanism that facilitates the expulsion of the As(III) from the microbial cell via phosphate transporters [39,156]. Consequently, through these complex mechanisms of redox transformation that cycle between As(V) and As(III) oxidation states [157], the dynamics of As in the environment are affected and often lead to the mobilisation of As into the surrounding. It is important to note that the prevailing conditions, such as the pH, ionic strength, energy source, substrate nature, availability of Fe(III)-oxides, and type of bacteria involved, are critical factors in the entire transformation process.

#### 6.1.1. Uptake of Nutrients from As-Bearing Minerals by DARB

Generally, microorganisms can dissolve and use different minerals as a source of nutrients and energy. Certain microbes often use minerals as their sole source of inorganic substrates such as carbon, nitrogen, and phosphorus for energy generation [9]. Others utilise sulphate (SO_4_^2−^), O_2_, Fe(III), and organic material as terminal electron acceptors. However, some sulfide minerals serve as a reservoir of enzyme cofactors, for example, copper (Cu), zinc (Zn), molybdenum (Mo), magnesium (Mg), and apatite often serve as the source phosphorus required for deoxyribonucleic acid (DNA), ribonucleic acid (RNA), adenosine triphosphate (ATP), and phospholipid synthesis [158].

Several scholars deliberated on the mechanism evolved by microorganisms to absorb nutrients from minerals. These comprise the primary colonisation process, which includes the mechanical infiltration of the rock surface, leading to mineral disintegration [159] and the subsequent dissolution of the mineral facilitated by organic agents produced by the cells (Figure 2) [9]. To accomplish this, bacteria typically secrete exopolysaccharides (EPS), which play an essential role in mineral colonisation and protect the bacteria from environmental stresses such as heavy metal toxicity. Drewniak and Sklodowska [9] stated that the physical disruption of the minerals occurs due to the production of EPS and its binding to the exposed mineral surface. Similarly, Welch et al. [159] previously highlighted the role of the EPS as a substrate for other (heterotrophic) bacteria producing organic agents, which in turn stimulate the chemical dissolution of the underlying minerals. However, some bacteria use an indirect approach of As(V) dissolution [160], which is achieved through the biosynthesis of a protein called a siderophore. This protein usually secretes small organic metal chelators and captures insoluble Fe(III).

Essentially, some bacteria use siderophores to survive in harsh environmental conditions or as organic ligands and/or acids that may facilitate the release of nutrients in the surrounding environment when essential nutrients for growth are limited or absent [9,161]. Interestingly, these organic ligands can establish strong complexes with metal cations and bind to mineral surface atoms. For these reasons, siderophores have been among the most common natural bacterial agents involved in mineral dissolution. Furthermore, these compounds have the power to enhance the transfer of an electron to Fe(III); hence, when they form strong complexes with Fe(III), this increases the solubility, resulting in the mobilisation of the bound metals [15,162].

Siderophores are typically secreted by DARB strains outside of their bacterial cell envelopes. Notably, these secretions have a high affinity for metal ions and are capable of chelating not only As but also Fe, Mn, Mg, Zn, Cr, uranium (U), gallium (Ga), nickel (Ni), Pb, and Cd [163,164,165]. As a result, these siderophores may help to reduce As bioavailability and subsequent removal from contaminated environments [166,167]. In another study by Lukasz et al. [8], it was established that siderophore production could enhance the dissolution of secondary As-bearing minerals. This observation was based on a biochemical test performed on DARB samples from the ancient Zloty Stok gold mine, which revealed that under iron-limiting conditions, all of the sampled DARB isolates developed siderophores known as hydroxamates, which were actively involved in the dissolution of As-containing minerals [8].

#### 6.1.2. Dissolution of As-Bearing Minerals by DARB

The reductive dissolution of the parent As-bearing minerals usually results in the release of As into the environment. Under reducing conditions, arsenate adsorbed to the surface of Fe minerals (e.g., scorodite or ferrihydrite) is used mainly by bacteria as a terminal electron acceptor in As reduction [9,31,43,168,169]. As a result of this process, dislodgment of attached arsenate and its subsequent mobilisation into the environments from secondary minerals occur (Figure 2). However, it is worth mentioning that, occasionally, chemoautotrophic arsenite-oxidising bacteria (CAO) makes use of oxygen and sometimes nitrogen as their terminal electron acceptor during the fixation of inorganic carbon (CO_2_) into cell material to oxidise aqueous As(III) to As(V).

In essence, the reduction of As(V) to As(III) is regarded as an efficient resistance and coping mechanism demonstrated by most DARB against the toxic effect and high arsenic concentration in the surroundings. In the same way, the process serves as the most accessible route through which DARB mobilise aqueous As(III).

To date, a wide range of bacterial species that play a key role in the redox transformations and cycling of As in the environment have been reported [64]. However, because of the differences in genomic contents, substrate types, modes of interactions with prevailing environmental conditions, and relationships with organic and inorganic fractions, including various (hydro)oxides [36,82], the action mechanism of different bacterial strains differ. For example, *Desulfuribacillus alkaliarsenatis* is a microbial strain with a broad dissimilatory metabolic capacity, which, apart from arsenate, can also grow using H_2_ pyruvate, formate, or lactate as an electron donor [170]. Furthermore, the *Desulfurispirillum indicum* S5 strain was reported to utilise multiple reductive pathways for arsenate and other elements such as selenate (SeO_4_^2−^), selenite (SeO_3_^2−^), nitrate (NO_3_^−^), and nitrite (NO_2_^−^) [171]. Another important bacterial strain that plays a vital role in the mobilisation of arsenic through dissolutions of As-bearing minerals is *Shewanella oneidensis* MR-1 [40,64,118,172,173,174,175,176].

### 6.2. Heterotrophic Arsenite-Oxidising Bacteria (HAO)

It is a fact that some bacteria are recognised for their ability to oxidise organic carbon using available oxygen as a terminal electron acceptor for energy generation. Using a similar process, some of these bacteria obtain their energy through As(III) to As(V) oxidation. Hence, they are regarded as HAO [177]. Notably, some of these bacteria are mixotrophic, meaning they obtain energy from organic carbon oxidation and As(III) to As(V) oxidation [177,178]. Interestingly, this process helps to detoxify As in the environment [179,180] by reducing the bioavailability of toxic As to the surrounding environment and plants [181].

### 6.3. Chemoautotrophic Arsenite-Oxidising Bacteria (CAO)

While some bacteria obtain their energy for metabolism through the oxidation of organic molecules, other bacterial species derive their energy from the oxidation of inorganic energy sources such as carbon monoxide, hydrogen, nitrogen and sulfur compounds, or divalent cations (e.g., Mn^2+^ and Fe^2+^) [182,183]. Similarly, some bacteria employ the same mechanism to obtain their energy during As(III) oxidation autotrophically. Hence, they are regarded as CAO [179,180]. A good example is the *Thermus* strain HR13, which has been shown to oxidise As(III) to As(V) under aerobic conditions while using arsenate as the terminal electron acceptor under anaerobic conditions [184]. It is worth noting that, in contrast to HAO which require the support of organic matter for their growth, the growth of CAO can be sustained through the oxidation of As(III) [185]. For this reason, most of the As(III) oxidation by HAO is widely considered a detoxification mechanism [179,181,185].

In addition to lessening the toxic effects of As, the presence of these As(III)-oxidising bacteria is believed to promote the activity and growth of some indigenous microbial communities in arsenic-contaminated soils, which subsequently enhances the As uptake efficiency. This attribute is demonstrated by *Ensifer* sp. M14, an As(III)-oxidising bacteria when added to As-contaminated soil [181]. According to the findings, exposure to bacteria promotes As uptake by the plant *Medicago sativa* (alfalfa) and reinforces the activities and growth of the microbiota [181,186].

## 7. Removal and Detoxification of Transformed Arsenic

**Figure 3:**
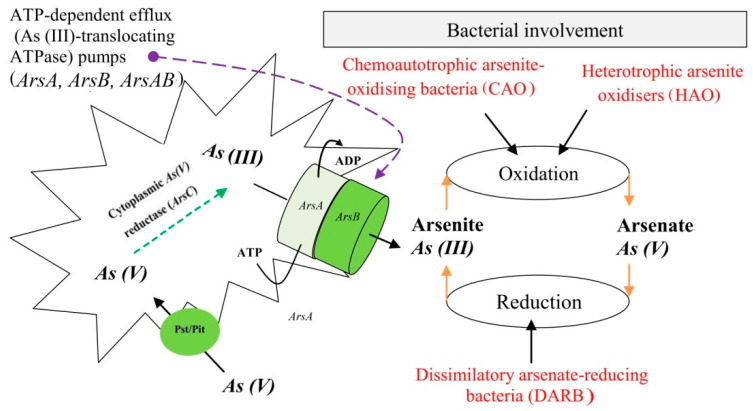
Conceptual diagram describing the reductive dissolution of As(V) to As(III) under the influence of the arsenic reductase enzyme, which facilitates the release of As(III). On the other side, chemoautotrophic and heterotrophic oxidising bacteria oxidise the release of As(III) into As(V). Dissimilatory arsenate-reducing bacteria (DARB) reduce available As(V) into As(III). Pst and Pit are the respective high-affinity (low-capacity) and low-affinity (high-capacity) phosphate (PO_4_^3−^) transporter systems responsible for As(V) intake. Courtesy of conceptual diagram: Aminu Darma and Peiman Zandi.

As pointed out earlier, bacteria have a different approach for eliminating As from the environment. DARB utilise a protein domain called arsenate reductase protein “*ArsC*” to enzymatically remove As in the environment [22]. *ArsC* is a small protein (13–16 kilodaltons) located in the cytoplasm of the bacteria facilitating the reduction of As(V) to As(III) [105,187]. In the process, glutaredoxin, glutathione or thioredoxin serve as an energy source for this transformation process [22,188]. At the end of this process, a flux of toxic As(III) is observed, mediated by an ATP-dependent efflux pump, *arsB* (Figure 3). Because of the integral role of ATP in this process as an essential energy source, under an anaerobic state, DARB acquires energy through redox transformation of As(V), where it is used as an electron acceptor instead of oxygen [22]. Notably, among many groups of bacteria, ε-Proteobacteria comprising *Sulfurospirillum arsenophilum* and *Sulfurospirillum barnesii* were the first DARB identified in the mid-1990s, and their representatives have since been recognised in other phylogenetic groupings, including δ- and γ-Proteobacteria and the low-GC “Gram-positive” bacteria, Crenoarchaea, and thermophilic eubacteria. Understanding the mechanism by which these bacteria detoxify and eliminate transformed As from their cells is critical to understanding the role of DARB in As transformation, toxicity, and dynamics in different settings.

### 7.1. Mechanism of Arsenic Detoxification from DARB Cells

As a consequence of the transformation of As(V) to As(III) by DARB and subsequent availability of As(III) in the bacterial cell, some key biochemical processes may be obstructed in the organisms [48,106]. This is because trivalent arsenic (As(III)) is more toxic than As(V) [189], primarily due to its ability to react readily with sulfhydryl and thiol (R-SH) groups in protein residues of the cell [106,189]. To minimise the excessive toxicity of this As species, the bacterial cell has to devise a coping mechanism to either (a) eject or expel the As(III) from the cell or (b) undergo methylation of As(III) to trivalent organoarsenicals [21,96,106,189].

#### 7.1.1. Ejection or Expulsion of As(III) from the Cell

As previously stated, in the transformation of As(V), the As(III)-specific transporter (*arsB*) gene mediated by *arsC* usually facilitates the expulsion of the transformed As(III) from the microbial cell (Figure 2 and Figure 3) [190].

Generally, the detoxification pathways employed by DARB against the toxic effect of As(III) are regarded as a resistant mechanism that is controlled by an arsenic resistance (*ars*) gene [106]. This *ars* gene is mostly encoded on the operon of the plasmid or chromosome in gram-negative or gram-positive bacteria [105,106,119,187,191]. Most DARBs’ detoxification process of inorganic arsenicals is controlled by *ars* operons; however, to efficiently detoxify organoarsenicals, the *ars* gene occasionally associates and combines with other *ars*-related genes.

The expulsion or extrusion of As(III) by most DARB, as their basic detoxification mechanism, is controlled by the *arsB* gene encoded in the *ars* operon [106,189]. Even though the nature of the *arsB* as membrane transport protein has allowed it to accomplish the role of catalytic expulsion of As(III) from the cell via membrane potential alone [192], sometimes it does coexpress with another *ars* operon protein *arsA* to form a tight membrane *arsAB* complex that provides a greater level of resistance (Figure 3) [106,189]. Also, because the *arsA* gene encodes the *arsA* protein as an As(III)-stimulated ATPase, the complex formed becomes an ATP-driven efflux system [189,193,194]. This *arsA* is distributed in the periplasm and is composed of functional homodimers with identical halves, each containing ATP binding sites, which serve as the catalytic component of the *ars* pump [106,195].

Other key important *ars* genes encoded in the *ars* operon that enhance the expulsion of As(III) are the *arsR* and *arsD* genes. The former detects inorganic arsenic in the environment and negatively regulates the transcription of other *ars* operon genes [196], while the latter functions as an As(III) metallochaperone, facilitating As(III) transfer to the metal-binding region of *arsA* of the *ars* efflux pump [197,198]. Because of the interaction of these genes, the overall ability of *arsB* to expel As(III) is enhanced due to the greater affinity of *arsA* for As(III) and the surge in ATPase activity of *arsA* even at lower As(III) concentrations [106,199].

#### 7.1.2. As(III) Methylation to Trivalent Organoarsenicals

Increasing evidence suggests that the methylation process functions as an additional system that is critical in the detoxification of As(III) [104], and it can also serve in the bioremediation of As [48]. The process involves adding a methyl group to As(III) and subsequent detoxification of methylated products [75,81,105]. According to Qin et al. [48], arsenic methylation involves two pathways, one involving reductions of As(V) by reduced glutathione tablets within the microbial cell and combining it to a free methyl donor [149], while the other involves a direct combination of the arsenic ion with a methyl donor [48,200]. Thus, the methylation processes comprise the reduction of As(V) to As(III), followed by the sequential coupling of As(III) with positively charged carbon ions in free methyl donors to generate MMA, DMA, and TMA as products [47].

**Figure 4:**
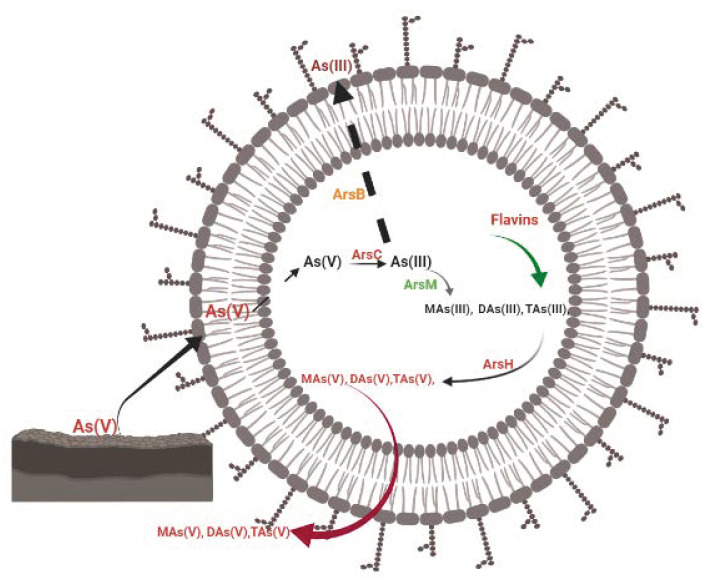
The description of how As(V) from the environment enters bacterial cells. Within the bacterial cell, As(V) is reduced to As(III) under the influence of *ArsC*. The *ArsB* gene then facilitates the expulsion of the available As(III) from the cell, while the action of *ArsM* aids in the methylation of some toxic inorganic As(III) into methylated organoarsenicals. Further action of the *ArsH* gene within the bacterial cell leads to the formation of less harmful methylated organoarsenicals, which can be eventually ejected from the bacterial cell. Courtesy of proposed model: Aminu Darma.

The methyl group is transferred to As(III) by the activity of As(III) S-adenosylmethionine methyltransferase, which is catalysed by *arsM* encoded in the *ars* operon (Figure 4) [48]. At the end of the process, *arsM* transform As(III) into dimethyl arsenate (DAs(V)) [201], trimethyl arsenite (TAs(III)), dimethyl arsenite (DAs(III), and methyl arsenite (MAs(III)) [48,104,106,202].

Although some organoarsenicals are harmful, the toxicity of As compounds are still reduced by methylation, which can be considered another defensive mechanism against arsenic toxicity by some bacteria [47,203]. Prospectively, this process could be a valuable strategy to minimise the availability of toxic arsenic in the environment. Bacterial strains such as *Aeromonas* sp., *Flavobacterium* sp., *Proteus* sp., *Pseudomonas* sp., *Rhodopseudomonas palustris*, *Corynebacterium* sp., *Escherichia coli*, *Achromobacter* sp., *Alcaligenes* sp., and *Nocardia* sp. have demonstrated the ability to produce methylated arsenic compounds [149]. Furthermore, recently, in an attempt to evaluate the impact of methylation on arsenic by *S. oneidensis* MR-1, the bacteria were exposed to 1.0 mg/L of As(V). The results indicate that the bacteria could tolerate low concentrations of inorganic As by methylating it into less toxic and organic DMA [47].

## 8. Contribution of *Shewanella oneidensis* MR-1 to As Redox Cycling and Remediation

It was highlighted that *Shewanella oneidensis* MR-1 is an effective bacterial strain contributing significantly to redox cycling and arsenic remediation. This role is accomplished by reducing Fe mineral-containing As and total or partial transformation of some toxic arsenicals into non-toxic equivalents. Hence, it is vital to understand this bacterial strain’s biology, genomic composition, and mechanism of arsenic transformation.

### 8.1. Biology and Distribution of Shewanella Genus

*Shewanella* was initially assigned to the *Achromobacter* genus but was later reclassified as prokaryotic and assigned to the *Shewanella* genus [204], which at present contains about 70 species and more than 30 available sequenced genomes isolated from deep-sea sediments, freshwater sediments, and other food sources [46,88,205]. Free-living strains thrive under varying temperatures, pressure, salinity, and nutrient compositions [206,207]. Some of them are associated with other organisms as epibionts, syntrophs, symbionts, and pathogens [138,208]. Interestingly, it could perfectly fit into redox-stratified environments due to its ability to adapt to and withstand different environments and associated stress [46,209,210,211]. Moreover, its physiology has supported its ability to survive in broad and different ecological niches from equatorial to polar regions, fresh water to oceans, the surface to subsurface sediment, and polluted to unpolluted environments [46,208].

In addition to the ability to occupy a wide range of environments, as pointed out earlier, the *Shewanella* genus has metabolic flexibility, which enables species of this genus to choose a mode of metabolism according to the immediate environmental conditions [206,212]. This genus is notable for its ability to respire using a variety of organic and inorganic materials, including oxidised metals such as Mn(III, IV) and Fe(III)*,* metal oxides such as hematite, magnetite, elemental sulphur (S^o^), thiosulphate (S_2_O_3_^2−^), sulphoxide, and arsenate, among others [205]. Notably, the genus possesses a high tolerance to numerous toxic compounds, allowing it to thrive in varying degrees of polluted environments and hazardous products. As a result, most strains in this genus play a positive bioremediation role [46]. Interestingly, *S. oneidensis* MR-1, a representative of this species, has been consistently involved in redox cycling and has been proven to be a valuable tool in the remediation of As-contaminated systems [21,47,148,213,214], accomplished through the previously mentioned reductive dissolution and detoxification mechanisms.

Another unique feature of this *Shawenella* genus from other prokaryotes is its role in the oxidoreduction cycle and its ability to survive in metal-rich environments [46,215]. This role is partly related to its remarkable ability to transfer electrons from the quinol pool to the external membrane’s outside face, which is facilitated by a complex assembly of cytochromes (c-type cytochromes) via a process called extracellular electron transfer (EET) [52,174,176,215]. Studies have shown that the EET process or pathway serves as an important bridge that enables electron transfer across the outer membrane through the periplasm from the inner membrane [123,126,212]. Because the minerals associated with Mn(III/IV) or Fe(III) are insoluble at circumneutral pH [126,216], it is therefore highly unlikely for them to cross the outer membrane and the periplasm, where bacterial terminal reductases for other electron acceptors, such as SO_4_^2−^, O_2_, and NO_3_^−^, are situated.

Fe(III) reduction facilitated by DIRB is a critical process for As mobilisation in the environment [124,148], a process that contributes significantly to As contamination of various environmental components. Interestingly, electrons for the Fe(III) to Fe(II) reduction are typically derived from EET through the oxidation of quinol to quinone (QH_2_), which further enters cells through a phosphate transport channel mediated by QH_2_ dehydrogenases such as tetraheme cytochrome c (CymA) [217,218]. Lopez-Adams et al. [124] recently highlighted the significance of the EET process in the mobilisation of As(V). The study revealed how flavin from *Shewanella* sp. significantly facilitated the release of As(V) through the reduction of Fe(III)-(oxyhydr)oxide from the As(V)-bearing Fe(III) mineral. Moreover, the same study further demonstrated the role of these bacteria in the mobilisation of As(V), as opposed to the reduction of As(V) to As (III).

#### Extracellular Electron Transfer (EET)

Generally, EET is a process employed by microorganisms to exchange intracellular electrons with an extracellular electron donor/acceptor across the cell membrane [212]. This electron donor/acceptor may vary from naturally occurring metal compounds to artificial electrodes [219]. To ensure that electrons are effectively transferred from the inner to the outer membrane, organisms evolved a metal reduction pathway (Mtr), which is supported by an array of c-type cytochrome protein components such as CymA and outer membrane cytochromes OmcA, MtrC, and MtrA [126,212] as well as the porin-like trans-outer membrane protein MtrB [137,138]. The EET pathway has been described to include many interchangeable periplasmic electron carriers, such as fumarate reductase, i.e., flavocytochrome c3 (Fcc3) or the small tetrahaem cytochrome (STC), which are reduced by central quinol oxidase CymA [46,220].

In some *Shewanella* species, particularly *Shewanella oneidensis* MR-1, the EET can be assisted by two similar external membrane complexes; the metal-reducing systems MtrCAB and MtrDEF [46]. However, deletion experiments demonstrate that MtrCAB alone tends to function physiologically [212,216,221]. Broadly, each of the complexes comprises two decaheme cytochromes associated with an external membrane and a transmembrane β-barrel protein, forming a channel through the membrane’s outer layer [220].

The EET process can be carried out in one of three ways: direct, distant, or mediated contact (facilitated by external soluble electron carriers) [46,212]. Direct contact occurs when there is uninterrupted contact between the cells and the metallic acceptors, and the transfer of electrons is accomplished from the outer face of the outer membrane [222]. While in some cases, scarcity of electron acceptors causes the cell’s outer membrane to undergo dynamic structural alteration, forming an outer membrane extension called nanowires. This alteration enables connection and electron transfer between the bacterial cell and the distant electron acceptors through a distant contact process [175,222,223]. Indeed, this is important, especially in bacterial metal reduction biofilm setup, due to the barrier between upper-layer organisms and electron acceptors. In another situation, the mediated process can be achieved using secreted electron carriers such as flavin that serve in electron shuffling in the external media and as significant cofactors of the external metal-reducing systems [212,224]. However, it is important to note that most flavins (secreted electron carriers) are typically secreted in laboratory batch cultures when the concentration of the bacteria is high [46].

The importance of flavin was established based on demonstrations when it was secreted at the outer membrane of MR-1, influencing electron transfer through the electron shuttling pathway [122,216]. Although some researchers [38,107,141,225] were able to describe the detailed role of flavin in the shuttling of electrons in Fe(III) reduction in oxic settings, the mechanism by which flavin influences the reduction of Fe(III) by MR-1 has not been sufficiently explained by researchers.

**Figure 5:**
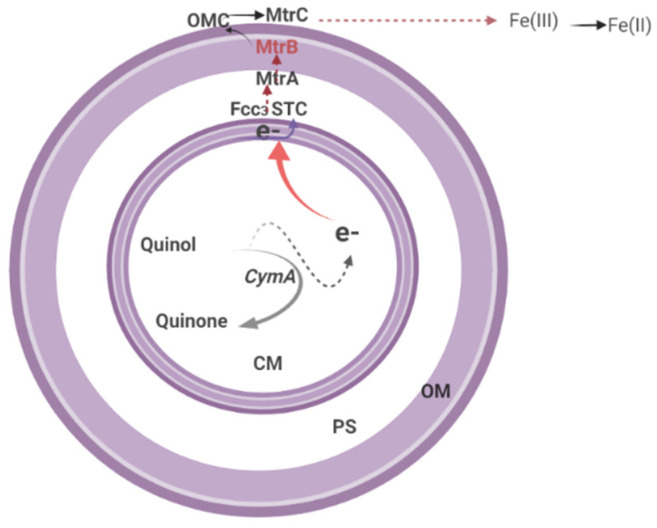
The suggested extracellular electron transfer (EET) for *Shewanella oneidensis* MR1. In the cytoplasmic membrane (CM) of the system, electrons are released through the oxidation of quinol to quinone. These electrons are then passed across the periplasm (PS) via flavocytochrome c3 (Fcc3) and tetraheme cytochrome (STC) periplasmic proteins. Through the action of electron shuffling systems such as flavin, MtrAB aids electron transfer across the outer membrane decahaem c-Cyts, i.e., MtrC and OmcA on the outer membrane (OM) of the bacteria from where it stretches to the extracellular Fe(III) to yield Fe(II). Courtesy of proposed model: Aminu Darma.

On most occasions, the exterior face of the outer membrane of the *Shewanella* genus receives electrons from the quinol pool, which is assisted by c-type proteins [38,212]. CymA, an inner membrane dehydrogenase, oxidises quinol and indirectly or directly transfers the released electrons to MtrC through periplasm proteins to reduce insoluble Fe(III) [217,226]. MtrA, in collaboration with MtrB, facilitates the transport of electrons to MtrC and OmcA, which are situated on the bacterium’s surface [227,228]. The direct or indirect reduction of Fe(III)-containing minerals by these bacteria, more specifically *MR-1*, is accomplished through the action of this outer membrane c-type (MtrC and OmcA) facilitated by the extracellular secretion of the bacterial cell such as flavin, which serves as an electron shuttling system (Figure 5) [126].

### 8.2. EET Process in Shewanella oneidensis MR-1

*Shewanella oneidensis* MR-1 (previously *Alteromonas putrefaciens*) is a facultative aerobic bacterium capable of dissimilatory reduction of manganese and iron oxides [137,229]. The bacterium is gram-negative in structure, with its cytoplasmic or inner and outer membranes on its cell envelopes separated by periplasm [230].

So far, the genomes of more than 200 strains in the *Shewanella* genus, including *Shewanella oneidensis* MR-1, have been sequenced, and comparative genomic studies have revealed that these bacteria share genetic makeup [205]. Genomic analysis of this bacterium has also shown that it comprises 47 two-component systems and 211 one-component systems, which demonstrated the complexity of the energy conversion pathways of the organism [206]. These components serve as the foundation for understanding the bacterial system’s sensing, signal transduction, and control. The system consists of networks of receptors, transmitters, and information regulators that link intracellular and extracellular signals to cellular responses [229].

Depending on the settings and the environmental conditions, MR-1 often has different forms of respiration. For example, it can exist as a facultative anaerobe that utilises Mn(III/IV)/Fe(III) as terminal electron acceptors or molecular oxygen (O_2_) for aerobic respiration [126,231]. These anaerobic and aerobic forms of respiration often result in the generation of Fe(II) and hydrogen peroxide (H_2_O_2_) [135,206,229,231].

The EET process in MR-1 is similar to the *Shewanella* genus process described above. Quinol dehydrogenase is located on the cytoplasmic membrane of MR-1, promoting the oxidation of quinol to quinone and subsequent transmission of the liberated electron to the periplasmic STC and Fcc_3_ for further reduction of Fe(III) (hydr)oxides (Figure 4) [107,232]. Principally, STC and Fcc_3_ relay the generated electrons across the periplasm to MtrA, which forms part of the trans-outer-membrane protein complex containing MtrC and MtrB [107,113].

Electron transfer under typical anoxic conditions is accomplished through flavin secretion by MR-1 cells, which enable electron transfer for Fe(II) mobilisation from MtrC and OmcA. In a set-up comprising MR-1, the secreted flavins can either serve as electron shuttles between MtrC/OmcA and Fe(III)(hydr)oxides or as cofactors that enhance Fe(III) (hydr)oxides to receive an electron from MtrC/OmcA [38,141]. However, in a situation where there is a distance between the Fe(III) (hydr)oxides and the bacterial cells, the electron-hopping mechanism facilitated by MtrC and OmcA through the outer membrane and periplasm extension of the cells serves as an alternate channel for this species to shuttle electrons [175,222,233]. It is also worthy to note that, although the functions of MtrDEF in EET are yet unascertained, the MR-1 genes for MtrABC and OmcA are always present in the same gene cluster as MtrDEF, the homologs for MtrABC [206,228,234]. Furthermore, MR-1 genomes have two additional MtrAB homologs involved in dimethyl sulfoxide reduction (DMSO) outside the cell [235,236].

MR-1 oxidatively degrades substances, including metals, in an aerobic setting, through an advanced oxidation process (AOP) called the microbially driven Fenton reaction [38,237]. This process has a significant impact on As behaviour, particularly in the As(V)-bearing Fe(III) mineral system. In the reaction, a continuous supply of O_2_ and Fe(II) is required, and to compliment this requirement, this species can continuously reduce Fe(III) to Fe(II) [15]. Apart from O_2_, the bacterium can also utilise NO_2_^−^, NO_3_^−^, S_2_O_3_^2−^, S^o^, DMSO, and fumarate as terminal electron acceptors for respiration [140]. In Fenton’s reaction, H_2_O_2_ is produced due to the addition of O_2_ [238]. Subsequently, the already liberated Fe(II) reacts with the generated H_2_O_2_ to form the hydroxyl radical (HO^●^) and hydroxyl ions (OH^−^). The former is significant in metal decomposition and biogeochemical cycling, including As [239,240,241]. This distinguishing feature is one of the attributes allowing MR-1 to play an indispensable role in the decomposition of environmental pollutants, including heavy metals. The overview of the Fenton reaction is represented in the chemical equations below.

Fe^2+^ + H_2_O_2_
→ Fe^3+^ + OH^−^ + HO^•^ (1)

Fe^3+^ + H_2_O_2_ → Fe^2+^ + HO_2_^•^ + H^+^ (2)

H_2_O_2_ + HO^•^ → HO_2_^•^ + H_2_O (3)

HO_2_^•^ ⇌ O_2_^•−^ + H^+^ (4)

Fe^3+^ + HO_2_^•^ → Fe^2+^ + O_2_ + H^+^ (5)

Fe^3+^ + O_2_^•−^
→ Fe^2+^ + O_2_ (6)

Fe^2+^ + HO^•^ + H^+^ → Fe^3+^ + H_2_O (7)

Fenton reaction describes the formation of Fe^2+^ and hydroxyl radicals due to the coexistence of hydrogen peroxide H_2_O_2_ and Fe^3+^ [237]. Further reaction of Fe^2+^ and H_2_O_2_ produces OH^–^ and more hydroxyl radicals HO^●^ [238]. The HO^●^ serves as an important component for the degradation of As and other contaminants.

Recently, the influence of *Shewanella* sp. was evaluated to maximise HO^•^ generation as a driver of the Fenton mechanism that promotes the elimination of environmental contaminants. The results revealed that the bacteria could aggregate the formation of HO^•^ and the electrochemical generation of H_2_O_2_ [28]. Accordingly, this hypothesis provides significant insight into the potential for other strains in the *Shewanella* genus, perhaps MR-1, to perform a similar role, particularly in natural settings where heavy metal contamination is prevalent.

Recently, Wang et al. [30] evaluated the impact of the Fenton reaction on As sequestration in rice grains. The study observed that all rice treated with Fenton solution had a marked reduction in DMA, As(III), and As(V) concentrations in their tissues compared with those not treated with the Fenton solutions. The outcome of this research revealed significant iron plaque formation in the soil, which resulted in a decrease in the availability of As in rice tissue due to As adsorption on the iron plaque.

## 9. Flavin’s Contributions to the Detoxification and Sequestration of Toxic Arsenite

As previously described in the preceding sections, MR-1 is capable of mobilising Fe(II) and As(V) to the surroundings using flavin secretion during the EET pathway, particularly in an As(V)-bearing Fe(III) mineral setting. Additionally, it was demonstrated how it could facilitate the immobilisation of As due to iron plaque formation via Fenton’s reaction. However, the contribution of flavin to As’s fate can be explored in greater depth than just those functions. A study by Pi et al. [51] indicated that riboflavin (RBFH_2_), a flavin derivative, could promote the extracellular oxidation of As(III) under oxic conditions. In the study, a 22% increase in As(V) was observed from the initial As(III) concentration at pH values of 5.2, 7.0, and 9.0, which indicated that RBFH_2_ was actively involved in the oxidation of As(III) to As(V). Furthermore, the study identified that the As(III) oxidation by RBFH_2_ was H_2_O_2_ driven at pH 9.0 and HO^•^ driven at pH values of 5.2 and 7.0. This finding implied that Fenton’s reaction plays a crucial role in a wide range of environmental pH values, which could serve as a promising tool for an in situ control strategy for As mobility. However, it is worth mentioning that the above findings did not occur under the influence of bacterial strains serving as the primary source of the flavin; therefore, more research is required to precisely determine the role of flavin in bacterial consortia in a natural environment.

Another study by Yan et al. [106] further elucidated the role of flavin in the oxidisation of toxic trivalent methylated arsenicals such as trimethylarsenite (TMAs(III)), dimethylarsenite (DMAs(III)), and methylarsenite (MAs(III)) to less toxic pentavalent methylated arsenicals such as trimethylarsenic oxide (TMAsO(V)), dimethylarsenate (DMAs(V)), and methylarsenate (MAs(V)) (Figure 4). The oxidation of these methylated arsenicals was speculated to be facilitated by *arsH*. This idea was later confirmed when *arsH* catalysed the oxidation of MAs(III) to less-toxic MAs(V) [242,243]. The *arsH* gene, as an *ars* operon, has previously been linked to many processes, including the reduction of quinones [244], Cr(V) to Cr(III), and Fe(III) to Fe(II) [106,245]. Similarly, an interesting study by Huijbers et al. [242] on *Yersinia enterocolitica* revealed that the *ars* operon coding *arsH* facilitated the oxidation of toxic trivalent aromatic arsenicals to less-toxic pentavalent species, e.g., MAs(III) to MAs(V). These findings implied that *arsH* could promote simultaneous oxidation-reduction reactions in bacteria. However, the most important and pressing concern is whether MR-1 can mimic the same phenomena under natural conditions, which only further research can answer.

## 10. Concluding Remarks

Since arsenic toxicity is still a serious issue affecting various biosphere components, an effective and eco-friendly strategy for its mitigation is required. This study explores As toxicity, availability, and how the reduction mechanism of Fe(III) to Fe(II) by model DIRB (*Shewanella* genus) affects its biogeocycling and redox transformation in the environment. From the articles reviewed, *Shewanella oneidensis* MR-1, a model Fe-reducing bacterium, has a significant influence on As mobility and behaviour as a result of As(V) desorption caused by the transformation of Fe(III) to Fe (II) facilitated by the extracellular electron transfer process (EET). Interestingly, during the EET process, the same bacteria promote flavin secretion, which could enhance the formation of iron plaques through Fenton’s reactions.

Considering the detoxification mechanism of As through methylation pathways, which reduces the amount of toxic As in the environment, the importance of flavin in the oxidation of some toxic arsenic species, and, most importantly, the affinity of As for iron plaques, the *Shewanella* genus and its associated species can be considered a very promising tool for reducing in situ As toxicity and mobility, rather than simply agents that promote the release of As into the environment. This unique strategy, if implemented, has the potential of lessening phytoavailable As due to its ability to detoxify and/or sequester As on iron plaques, thereby reducing the overall toxic effect of As in the ecosystem. In this regard, a thorough understanding of the molecular mechanisms underlying MR-1’s multifaceted role in Fe(III) reduction, As detoxification, methylation, and sequestration will complement efforts to reduce the toxic effects of As in the environment.

## 11. Future Direction

More research is needed to improve *Shewanella*’s ability to efficiently transform arsenic in natural settings, as the process appears to be slow based on data from relevant literature. Integrating molecular approaches will significantly deepen the understanding of how the methylation process by MR-1 enables As detoxification, particularly in different environmental conditions. In addition, studying the detailed action mechanism of *ArsH* in MR-1 would provide more potential for the development of As remediation strategies.

Previous research has shown that the purification and characterisation of some vital proteins in microbial EET, particularly those involved in MR-1, contribute to a general understanding of the molecular basis for microbial EET. Hence, the biochemical characterisation of newly discovered microbial proteins (e.g., MtrDEF) directly involved in EET will undoubtedly contribute to a better understanding of the molecular mechanisms of MR-1 EET.

Most flavins are secreted in laboratory batch cultures when the bacterial concentration is high. However, not much information is available in the literature on the availability and effect of flavins on the redox transformation of As in a natural setting. Thus, future studies in such a direction are strongly recommended, especially since the presence of flavin even at a sub-micro-scale could have an impact. In addition, more studies are needed to support the idea that flavin concentrations in bacterial physiological biofilms enhance the distant contact mechanisms for EET. It is also critical to understand how flavins strengthen the iron oxide mineral or electrode reduction rate, especially when *Shewanella* sp. is present as the principal source.

Fenton’s reaction appears to be a promising solution for decreasing As uptake and translocation in rice and other crops. Hence, more emphasis should be placed on evaluating the Fenton mechanism in the presence of MR-1 consortia in a natural environment where the required components for Fenton’s reaction are present. Most studies on the ability of *Shewanella* spp. to drive Fenton’s reaction for pollutant degradation, including As, are still in the experimental stage; thus, field trials involving other species of *Shewanella* sp., other than MR-1, should be designed.

## 12. Limitations

Given that the microbial transformation process, particularly in natural settings, is slow, the approach described in this article may be time-consuming, hence constituting part of the approach’s limitation. Furthermore, because most *Shewanella* bioremediation strategies are conducted in a laboratory, the majority of the examples reported are laboratory-based, with only a few demonstrated in a natural setting.

Because each heavy metal has a distinct behaviour, and some bear different oxidation states relative to prevailing environmental variables, the article could not address the significance of *Shewanella* spec. for phytoavailability and toxicity of heavy metals in general.

## Data Availability

No associated data marked.
